# Toilet Paper, Minced Meat and Diabetes Medicines: Australian Panic Buying Induced by COVID-19

**DOI:** 10.3390/ijerph18136954

**Published:** 2021-06-29

**Authors:** Teyl Engstrom, Dolly O. Baliunas, Benjamin P. Sly, Anthony W. Russell, Peter J. Donovan, Heike K. Krausse, Clair M. Sullivan, Jason D. Pole

**Affiliations:** 1 Centre for Health Services Research, The University of Queensland, Herston, QLD 4006, Australia; t.engstrom@uq.edu.au (T.E.); Benjamin.Sly@health.qld.gov.au (B.P.S.); Anthony.Russell2@health.qld.gov.au (A.W.R.); clair.sullivan@health.qld.gov.au (C.M.S.); 2 School of Public Health, The University of Queensland, Herston, QLD 4006, Australia; d.baliunas@uq.edu.au; 3 Dalla Lana School of Public Health, The University of Toronto, Toronto, ON M5S 1A1, Canada; 4 Princess Alexandra Hospital, Woolloongabba, QLD 4102, Australia; 5 Royal Brisbane and Women’s Hospital, Herston, QLD 4029, Australia; Peter.Donovan@health.qld.gov.au (P.J.D.); Heike.Krausse@health.qld.gov.au (H.K.K.); 6 Faculty of Medicine, The University of Queensland, Herston, QLD 4006, Australia

**Keywords:** health services research, routinely collected health data, COVID-19, diabetes, health behaviour

## Abstract

The COVID-19 pandemic has impacted the management of non-communicable diseases in health systems around the world. This study aimed to understand the impact of COVID-19 on diabetes medicines dispensed in Australia. Publicly available data from Australia’s government subsidised medicines program (Pharmaceutical Benefits Scheme), detailing prescriptions by month dispensed to patients, drug item code and patient category, was obtained from January 2016 to November 2020. This study focused on medicines used in diabetes care (Anatomical Therapeutical Chemical code level 2 = A10). Number of prescriptions dispensed were plotted by month at a total level, by insulins and non-insulins, and by patient category (general, concessional). Total number of prescriptions dispensed between January and November of each year were compared. A peak in prescriptions dispensed in March 2020 was identified, an increase of 35% on March 2019, compared to average growth of 7.2% in previous years. Prescriptions dispensed subsequently fell in April and May 2020 to levels below the corresponding months in 2019. These trends were observed across insulins, non-insulins, general and concessional patient categories. The peak and subsequent dip in demand have resulted in a small unexpected overall increase for the period January to November 2020, compared to declining growth for the same months in prior years. The observed change in consumer behaviour prompted by COVID-19 and the resulting public health measures is important to understand in order to improve management of medicines supply during potential future waves of COVID-19 and other pandemics.

## 1. Introduction

COVID-19 has had an impact on the treatment and management of noncommunicable diseases in health systems globally [1]. Research indicates that diabetes is a risk factor for acquiring COVID-19 and people with diabetes are more likely to suffer severe illness from COVID-19, compared to those without [2]. The pandemic has interrupted management of diabetes in many jurisdictions, with studies in Italy reporting a significant drop in outpatient diabetes clinic visits for patients with type 2 diabetes, as well as a reduction in new diabetes diagnoses among children [3,4]. Studies of people with diabetes in India and Nigeria have highlighted issues with accessing their usual medicines during the pandemic [5,6]. Some clinicians in Australia have reported postponing non-urgent new diabetes appointments with unknown consequences for the patient [7].

Access to prescribed medications is important in reducing morbidity for people with diabetes [8]. While the pandemic has led to panic buying and subsequent shortages of consumer goods like toilet paper [9,10] and minced meat [11,12], it is unclear what impact a global pandemic may have on diabetes medication demand across the world. Australia offers an opportunity to study this, with readily available dispensed medicines data and is of interest as it imports over 90% of medicines—hence is highly dependent on global supply chains [13]. Therefore, the aim of this study was to explore the number of government subsidised diabetes prescriptions dispensed in Australia throughout the COVID-19 pandemic to assess any changes in behaviour. Future pandemics are imminent [14,15]; this research will help guide policy makers and clinicians to improve diabetes medicines management when the next pandemic hits.

## 2. Materials and Methods

This study considered prescriptions dispensed under the Pharmaceutical Benefits Scheme (PBS), Australia’s government medicine subsidy program [16]. PBS is available to those enrolled in Australia’s universal health care system, Medicare, which covers those living in Australia who are Australian or New Zealand citizens, who hold or are applying for permanent residency and other small groups including temporary residents covered by a ministerial order [16]. The PBS subsidises medicines over a minimum threshold, with lower co-payments for those eligible for concession (those receiving other financial benefits from the government) and a ‘safety net’ which further reduces co-payments when out-of-pocket costs of medicines in a calendar year reaches a pre-determined level [16].

The PBS website contains publicly available data describing the number of prescriptions by PBS item code, patient category (general, concessional, veteran) and month, based on the date the drug was dispensed to the patient [17]. It details PBS prescriptions dispensed under Section 85 of the National Health Act 1953, which includes most prescriptions written by general practitioners and dispensed by community pharmacies and some prescriptions dispensed by hospital pharmacies [17]. This data includes medicines which are under the minimum co-payment threshold [16,17]. Data from January 2016 to November 2020 has been compiled.

This study focuses on PBS item codes corresponding to Anatomical Therapeutical Chemical (ATC) codes for drugs used in diabetes care (ATC2 code = A10) [18]. This comprises ‘Insulins and analogues’ (ATC3 code = A10A) and ‘Blood glucose lowering drugs, excluding insulins’ (ATC3 code = A10B), hereafter referred to as insulins and non-insulins respectively [18].

Data were analysed using R software version 4.0.2 (R Foundation for Statistical Computing, Vienna, Austria) and figures produced using the ggplot2 package [19,20]. The number of diabetes prescriptions dispensed each month were calculated and plotted over time, along with key dates from the spread of COVID-19 in Australia [21]. Data were then stratified by insulins and non-insulins and number of prescriptions were summed and graphed by month and year dispensed. Data were also stratified by concessional and general patient types and number of prescriptions summed by month for each group. Those in the repatriation benefits scheme were not included in the figure due to small volumes. Finally, the total number of diabetes related prescriptions dispensed from January to November in 2020 were calculated and compared to prescriptions dispensed in the same months during prior years. This comparison was also carried out for insulins and non-insulins.

## 3. Results

**Figure 1:**
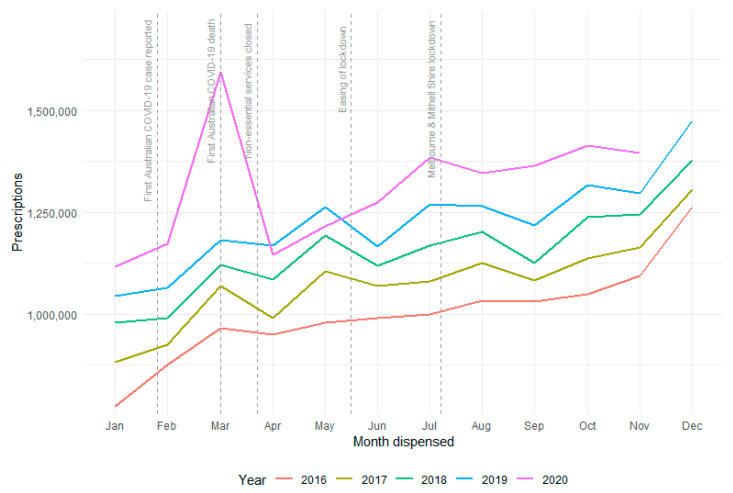
PBS (Pharmaceutical Benefits Scheme) dispensed diabetes medicines by year.

Between 2016 and 2019, monthly prescriptions of diabetes medicines dispensed through the PBS had been consistently increasing year-on-year by an average of 7.2% with a standard deviation of 2.4% (range: 3.4–13.9%), as shown in Figure 1. This trend continued in January and February 2020 with year-on-year growth of 6.9% and 10.3% respectively. In March 2020 there was a peak in the number of diabetes medications dispensed, at 1.59 million, up 34.8% on March 2019, which is 4.5 times the mean year-on-year growth (7.2% in 2016–2019). The March peak was followed by a drop in prescriptions dispensed during April and May 2020, 2.1% and 3.8% below the numbers seen in the corresponding months in 2019. From June 2020 onwards the number of diabetes medicines dispensed returned to levels in the range of previous year-on-year growth (6.4–9.4%), except for September which increased by 12.1% compared to 2019.

**Figure 2:**
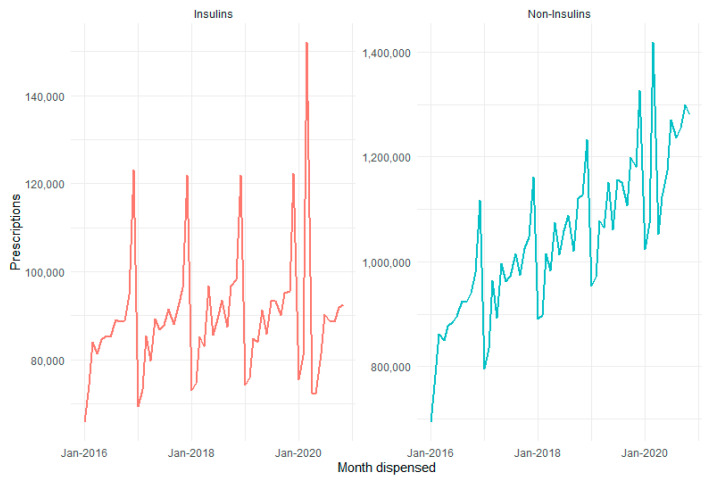
PBS dispensed diabetes medicines by insulins and non-Insulins.

The majority of diabetes medicines prescriptions dispensed in 2019 were for non-insulin medicines (93%), only 7% were for insulin medicines, as shown in Figure 2. The March 2020 peak and April–May drop was evident in both insulin and non-insulin diabetes medicines. These changes are much more pronounced in insulins, with 78.6% year-on-year increase in March 2020, a 50-fold-increase over the mean growth of 1.6% (2016–2019). Insulin prescriptions dispensed then fell 13.7% in April and 21.0% in May 2020 compared to the same months in 2019. While for non-insulins, March 2020 prescriptions dispensed were up 31.3% on March 2019 and then fell 1.1% and 2.4% in April and May respectively, compared to 2019.

**Figure 3:**
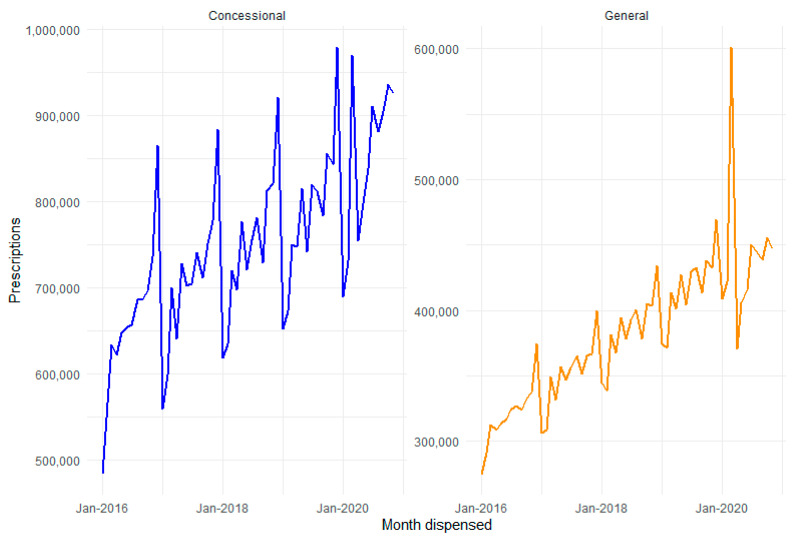
PBS dispensed diabetes medicines by patient category.

Concessional patients accounted for 64% of diabetes medicine prescriptions dispensed in 2019, 34% were general patients and 2% were for repatriation benefits scheme, as shown in Figure 3. A peak in prescriptions dispensed in March, followed by a drop in April–May 2020 was observed across all patient categories. The peak in demand was largest for the General patient category, with a 45.4% year-on-year increase in March 2020, compared to mean growth of 9.8% in previous years (2016–2019).

From January to November 2020 there were a total of 14.4 million prescriptions dispensed for diabetes medicines, an 8.8% increase on the same period in 2019, as shown in Table 1. This annual growth percentage is a small, unexpected increase compared to declining growth in prior years (8.3% in 2017, 7.2% in 2018, 6.3% in 2019). For insulin diabetes medicines there was a 2.3% growth of prescriptions from January to November 2020, compared to the same months in 2019, in the range of previous years’ growth (1.8% in 2017, 2.5% in 2018, −0.1% in 2019).

Monthly data from 2016 to 2019 follow similar patterns with number of prescriptions dispensed increasing throughout the calendar year, this is a known phenomenon due to the safety net effect [22].

## 4. Discussion

A large peak in diabetes medicines prescriptions dispensed in March 2020 was observed, likely the result of ‘panic buying’ or stockpiling as cases of COVID-19 in Australia rose from 26 to 4543 [23] in that single month and most Australian states and territories announced border closures [21]. Purchasing excess quantities in times of crisis is a well-documented phenomenon, known as panic buying, and can be motivated by expected future scarcity and a desire to exhibit control [24]. Australia had reduced COVID-19 cases in the following months, recording only 2386 new cases in April and 430 new cases in May [23]. The number of diabetes prescriptions fell below 2019 levels in April and May 2020, which is likely due to people having purchased excess supply in March, reduced doctors’ appointments, as well as increased government encouragement to stay home [23]. A larger COVID-19 outbreak occurred in one Australian state, Victoria, from July to September 2020, resulting in approximately 15,000 new infections [23], however state specific data were not available to monitor how this impacted diabetes medicines dispensed in Victoria. Australia has continued to fare well, recording less than 30,000 COVID-19 cases and less than 1000 deaths in 2020 [23].

Panic buying behaviour was more pronounced in General patients, compared to Concessional patients, which may be due to a lack of resources to purchase multiple prescription repeats at a time. This finding adds support to other studies which suggest the negative economic and health impacts of COVID-19 in Australia are disproportionately felt by already vulnerable populations [25]. A larger panic buying effect was evident for insulin medicines compared to non-insulins. This is likely appropriate, as the clinical consequences of missed insulin doses can be immediately harmful, whereas complications from missed non-insulin diabetes medications are delayed. The observed change in consumer behaviour prompted by COVID-19 and the resulting public health measures is important to understand to improve management of medication supply during potential future waves of COVID-19 and other pandemics. This is especially important for medicines for conditions like diabetes which have been highlighted as risk factors for COVID-19, potentially preventing people from seeking treatment in the usual way [2].

Dispensation of medicines through the PBS in Australia are highly regulated. Pharmacists are required to wait at least 4 or 20 days (depending on type of medicine) between dispensing repeats on prescriptions [26]. An exception to this rule can be made if a general practitioner determines that the patient needs more medicine than a single supply and that it would be difficult for them to obtain the medicines across multiple occasions [27]. In this case, they can write a Regulation 49 prescription which allows patients to receive original and repeat supplies at the one time [27]. During the COVID-19 pandemic, general practitioners issued more Regulation 49 prescriptions in an effort to help their patients isolate [28], unaware of the potential for hoarding behaviour or resultant pharmaceutical supply issues. This contributed to the peak in diabetes medication dispensed in March 2020, which depleted stocks in many pharmacies and wholesalers [29].

The Therapeutic Goods Administration (TGA) responded on 19 March by limiting pharmacies to dispense a one-month supply of both insulin and non-insulin diabetes medicines [29] regardless of Regulation 49 prescriptions. By April 2020, Diabetes Australia provided advice that there were no stock shortages and discouraged people from stockpiling medication, however shortages of metformin modified-release 500 mg were again reported in July [30,31]. Implementing purchase limits on diabetes medicines is similar to measures put in place for consumer goods in Australia early in the pandemic, with major supermarkets in Australia limiting the purchase of toilet paper from 5 March [32]. In normal times, the existing PBS regulation is sufficient to avoid medicine shortages, however this study highlights the need for proactive regulatory measures in times of crisis and improved education for clinicians and consumers to prevent shortages (Table 2).

In addition to updated TGA regulations, the Australian government also introduced measures to improve access to PBS medicines during the pandemic. ‘Continued dispensing’ policies enabled pharmacists to dispense medicines to patients without a current script, where they had previously been prescribed [33]. Pharmacists were also allowed to substitute appropriate medicines without approval from the doctor, where prescribed medicines weren’t available. A prescriptions delivery service was initiated for vulnerable patients, including people with diabetes, in order to reduce risk of COVID-19 exposure [34]. Electronic prescribing capabilities were also fast tracked [33]. These actions are in line with recommendations for management of prescriptions to treat non-communicable diseases during the pandemic, however a proactive rather than reactive response may have avoided temporary shortages and reduced risk of in-person contact sooner [35].

These findings are consistent with other jurisdictions. A study from Germany reported an 18% increase in insulins in March 2020, compared to March 2019 [36]. Studies from the US of commonly used medicines for non-communicable diseases have reported similar changing trends in prescriptions dispensed [37]. The Australian Institute of Health and Welfare published figures showing an increase in all PBS medicines dispensed across almost all ATC groups in March 2020, compared to March 2019 [38]. The peak in demand for diabetes medicines is also consistent with increased purchasing of consumer goods in Australia, such as minced meat and toilet paper. These goods are made in Australia and were able to respond to changes in demand by increasing production [32]; however, most medicines are imported to Australia [13], hence they are more vulnerable to shortages in times of global crisis.

There are some limitations of this study. The PBS data utilised in this study measures the number of prescriptions supplied, not individuals, hence we cannot determine whether everyone who required prescriptions got them, or if a small number of people stockpiled more medicine than necessary. The data do not distinguish between prescriptions for initial and continuing treatment, so we cannot make a judgement on whether COVID-19 has impacted the distribution of prescriptions for new or existing patients. Data is reported on a national level and hence response to variations in COVID-19 cases, lockdowns and logistical challenges by state could not be assessed.

## 5. Conclusions

This study found changes in demand of diabetes medications around the time of COVID-19 that is consistent with ‘panic buying’, leading to temporary shortages in Australian pharmacies and wholesalers in March 2020. Existing regulation is not sufficient to control medicines supply during times of crisis; governments and regulators should be proactive in utilising the policy levers available to them, such as limiting supply of medicines dispensed at pharmacies and implementing medication delivery for vulnerable people at the outset of future pandemics and disasters. Campaigns to improve both clinician and consumer knowledge of how best to manage diabetes medication in times of crisis are also needed. The frequency of emerging infectious diseases is likely to increase going forward, resulting in more pandemics [14,15]; the learnings from this study can be used to improve pandemic preparedness and protect those with diabetes.

## Figures and Tables

**Table 1:** PBS dispensed Diabetes medicines January to November by year.

Period of Supply	All Drugs for Diabetes (ATC2 Code = A10)		Insulin Drugs for Diabetes (ATC3 Code = A10A)		Non-Insulin Drugs for Diabetes (ATC3 Code = A10B)	
	Prescriptions Dispensed (*n*)	Year-on-Year Change (%)	Prescriptions Dispensed (*n*)	Year-on-Year Change (%)	Prescriptions Dispensed (*n*)	Year-on-Year Change (%)
January–November 2016	10,742,580	-	938,491	-	9,804,089	-
January–November 2017	11,633,312	8.3%	955,714	1.8%	10,677,598	8.9%
January–November 2018	12,473,017	7.2%	979,892	2.5%	11,493,125	7.6%
January–November 2019	13,260,953	6.3%	979,054	−0.1%	12,281,899	6.9%
January–November 2020	14,429,019	8.8%	1,001,276	2.3%	13,427,743	9.3%

**Table 2:** Summary of study findings and recommendations.

Finding	Recommendation
Significant increase in demand at outset of pandemic	Regulators should proactively introduce limits on purchasing Initiate campaigns to educate consumers about responsible purchasing behaviour
Panic buying was more pronounced for insulin medicines, compared to non-insulins, likely due to worse consequences of missing insulin doses	Regulators should ensure greater supplies of insulin medicines Purchasing limits should be enforced by pharmacists at an early stage of the pandemic to ensure all patients have access to critical medicines over time
Panic buying was more pronounced among General patients, compared to Concessional patients, likely due to limited purchasing resources	Regulators should ensure medicines are set aside for concessional patients
General practitioners issued more Regulation 49 prescriptions resulting in hoarding behaviour and consequent pharmaceutical supply/stock issues	Regulators should proactively introduce limits on purchasing Initiate an educational campaign for clinicians on the risk of consumer hoarding and medicine shortages
Consumers purchased more medicines than needed to avoid exposure to the virus	Regulators should continue to allow electronic prescribing Medicine delivery services should be continued to avoid the need for stockpiling

## Data Availability

The data presented in this study are openly available in https://www.pbs.gov.au/info/statistics/dos-and-dop/dos-and-dop, accessed on 15 March 2021.
